# Linking COVID-19 and Heme-Driven Pathophysiologies: A Combined Computational–Experimental Approach

**DOI:** 10.3390/biom11050644

**Published:** 2021-04-27

**Authors:** Marie-Thérèse Hopp, Daniel Domingo-Fernández, Yojana Gadiya, Milena S. Detzel, Regina Graf, Benjamin F. Schmalohr, Alpha T. Kodamullil, Diana Imhof, Martin Hofmann-Apitius

**Affiliations:** 1 Pharmaceutical Biochemistry and Bioanalytics, Pharmaceutical Institute, University of Bonn, An der Immenburg 4, D-53121 Bonn, Germany; mhopp@uni-bonn.de (M.-T.H.); milena.detzel@uni-bonn.de (M.S.D.); s6regraf@uni-bonn.de (R.G.); ben.s@uni-bonn.de (B.F.S.); 2 Department of Bioinformatics, Fraunhofer Institute for Algorithms and Scientific Computing (SCAI), Schloss Birlinghoven, D-53757 Sankt Augustin, Germany; Daniel.domingo.fernandez@scai.fraunhofer.de (D.D.-F.); yojanagadiya@gmail.com (Y.G.); alpha.tom.kodamullil@scai.fraunhofer.de (A.T.K.); 3 Enveda Biosciences, Inc., San Francisco, CA 94080, USA; 4 Causality Biomodels, Kinfra Hi-Tech Park, Kalamassery, Cochin, Kerala 683503, India

**Keywords:** biomarkers, cardiovascular risk, coagulation, COVID-19, heme, heme-binding motifs, hemolysis, inflammation, pathway networks, SARS-CoV-2

## Abstract

The SARS-CoV-2 outbreak was declared a worldwide pandemic in 2020. Infection triggers the respiratory tract disease COVID-19, which is accompanied by serious changes in clinical biomarkers such as hemoglobin and interleukins. The same parameters are altered during hemolysis, which is characterized by an increase in labile heme. We present two computational–experimental approaches aimed at analyzing a potential link between heme-related and COVID-19 pathophysiologies. Herein, we performed a detailed analysis of the common pathways induced by heme and SARS-CoV-2 by superimposition of knowledge graphs covering heme biology and COVID-19 pathophysiology. Focus was laid on inflammatory pathways and distinct biomarkers as the linking elements. In a second approach, four COVID-19-related proteins, the host cell proteins ACE2 and TMPRSS2 as well as the viral proteins 7a and S protein were computationally analyzed as potential heme-binding proteins with an experimental validation. The results contribute to the understanding of the progression of COVID-19 infections in patients with different clinical backgrounds and may allow for a more individual diagnosis and therapy in the future.

## 1. Introduction

At the beginning of 2020, the coronavirus disease 19 (COVID-19) was declared a pandemic of international concern and an unprecedented challenge for country-specific health care systems [1]. COVID-19 is caused by infections of severe acute respiratory syndrome coronavirus 2 (SARS-CoV-2) and accompanied by pneumonia, acute respiratory distress syndrome (ARDS) associated with a cytokine storm and death in the most severe cases [2,3,4]. The renin-angiotensin system (RAS), which is associated with hypertension, is directly associated with SARS-CoV-2 viral transmission [5,6,7]. The virus gains access to the host cell by docking of its spike proteins (S proteins) on the membrane surface of the host cell, which occurs via the transmembrane protein angiotensin-converting enzyme 2 (ACE2), an essential part of RAS [8,9,10]. ACE2 is expressed on the cell surface of alveolar epithelial cells of the lungs [11]. The interaction between S proteins and ACE2 involves several residues in the receptor-binding domain of the S protein and ACE2 that form hydrogen bonds, a hydrophobic interaction interface and a salt bridge [7,12,13]. Upon binding, the S protein is subjected to proteolytic cleavage by the host cell’s transmembrane serine protease subtype 2 (TMPRSS2) [8]. An interplay between the viral protein’s membrane protein (M protein), nucleocapsid envelope protein (E protein), and S protein may support the viral budding process [14,15]. Protein 7a acts as an accessory protein in virus–host interactions and virus particle formation, which is crucial before the release of reproduced virus particles into surrounding areas [16,17,18]. The immunomodulating functions of protein 7a through interaction with CD14^+^ monocytes has been demonstrated as well [19].

Numerous studies have provided information about the main symptoms, risk factors for severe disease progression, and clinical diagnostic values including blood routine, blood biochemistry, and infection-related biomarkers [2,20,21,22]. Among other criteria, the patients’ blood group seems to affect disease progression [23,24,25,26]. The main symptoms are fever, cough, and fatigue, all presenting reactions of an activated immune system [21]. The activation of the immune and the complement system is monitored by a variety of markers including increased values for interleukin (IL)-6 (52% of the patients), erythrocyte sedimentation rate (85%), serum ferritin (63%), and C-reactive protein (CRP) (86%) [2,20,27,28,29,30]. Furthermore, studies that monitored coagulation parameters of COVID-19 patients showed a tendency towards procoagulant states [22,31,32,33] and an increased risk of venous thromboembolism [34]. This was indicated, for example, by higher levels of fibrin/fibrinogen degradation products, fibrinogen, platelet hyperreactivity, increased neutrophil extracellular trap formation, and lower antithrombin levels [22,35,36]. In some patients, however, an increased bleeding risk was described, possibly occurring due to the presence of elevated tissue-type plasminogen activator [37]. Levels of hemoglobin and heme-scavenging proteins (i.e., hemopexin, albumin) were often changed in COVID-19 patients [2,20,21,29,38]. These clinical parameters are interrelated when viewed from the perspective of heme and its interaction radius [39,40,41,42,43]. Heme is the prosthetic group of hemoglobin that is responsible for oxygen transport in the blood [44]. Under hemolytic conditions, such as sickle cell disease (SCD) and β-thalassemia, hemoglobin is degraded, resulting in an accumulation of labile heme [44,45]. As a consequence, the heme-scavenger hemopexin, yet also albumin, become saturated, which allows heme to act as a cytotoxic, procoagulant, complement-activating and proinflammatory effector [39,41,43,46,47]. These responses are, in part, mediated through interaction of heme with proteins (e.g., tumor necrosis factor α (TNFα), Toll-like receptor 4 (TLR4) [41,42,48,49]) by upregulation of cytokines (e.g., IL-1β, TNFα) or through ROS-dependent induction of signaling pathways (e.g., MAPK/ERK pathway, NF-κB signaling) [39]. Although these findings suggest a link between processes implicated in SARS-CoV-2 and those related to heme, there is still a lack of information and data. Despite the recent efforts invested in research on SARS-CoV-2, knowledge of the molecular mechanisms responsible for the pathophysiology of the infection still remains scarce. Likewise, data concerning heme biology are underrepresented in bioinformatics resources such as pathway databases. This knowledge gap can be addressed by using custom-made models such as knowledge graphs (KGs) [50]. Placing research data in the right context by employing KGs supports the elucidation of biochemical mechanisms, the generation of novel hypotheses, and the identification of targets for drug repurposing [50,51,52,53]. Two of our recent scientific publications focused on KGs around heme as well as COVID-19 [42,54]. Although both studies are tangential, the generated KGs can subsequently be employed to investigate the overlap between hemolytic disorders and COVID-19. Therefore, two approaches were applied herein to explore a potential link between COVID-19 and heme-driven pathophysiology. We provide insights into pathways that might play a role when considering heme in the context of COVID-19 infections by superimposing the heme KG [42] and the COVID-19 KG [54]. Furthermore, we investigated the interaction of heme with select SARS-CoV-2 proteins and specific host cell proteins by applying our established strategy employing the heme-binding motif (HBM) prediction system HeMoQuest [55] and experimental studies to characterize the computationally predicted heme-binding sites in the viral and host proteins.

## 2. Materials and Methods

### 2.1. Modeling the Interplay between SARS-CoV-2 and Heme

**Figure 1:**
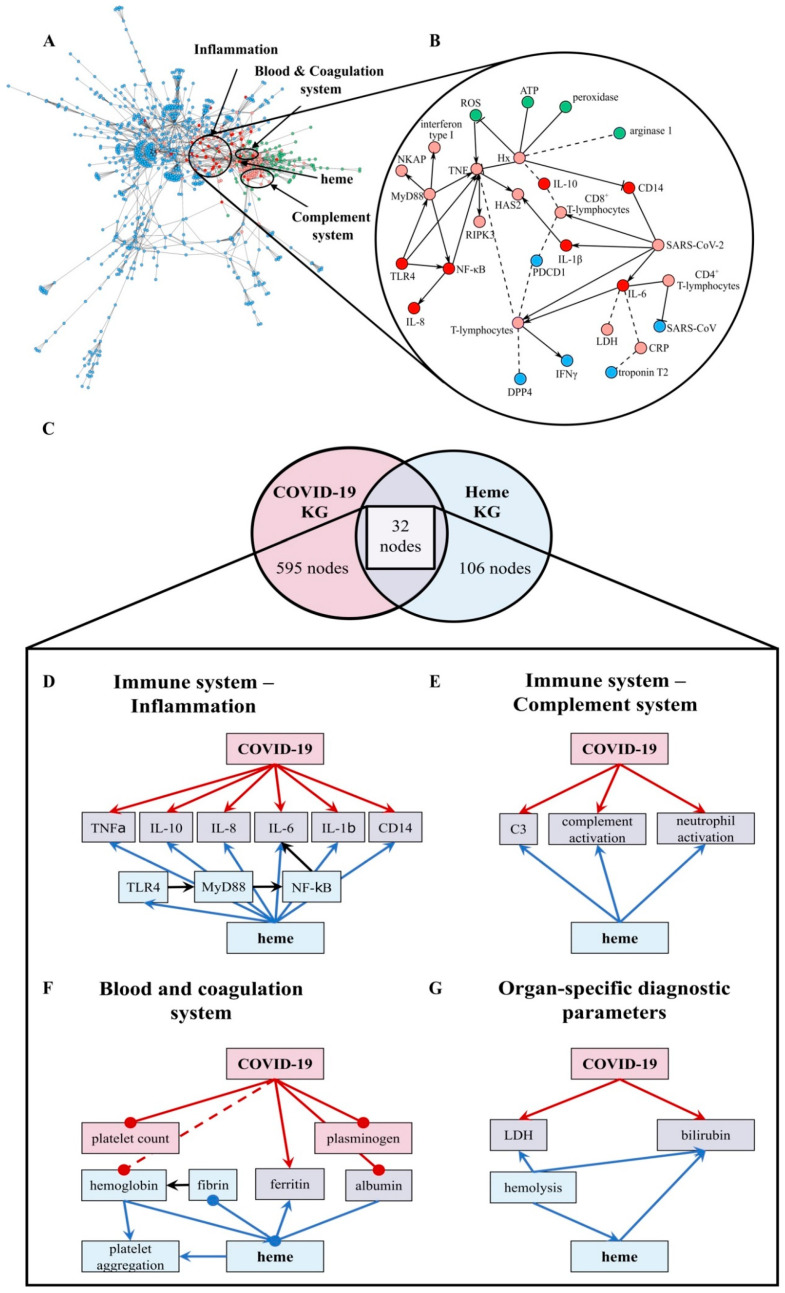
The overlap between the COVID-19 KG [54] and Heme KG [42] reveals shared biochemical pathways. (**A**) The network displays the largest area of overlap between the two KGs. Node coloring denotes whether a particular entity is present exclusively in the COVID-19 KG (blue), in the Heme KG (green), or in both (red). Neighbors of overlapping nodes are colored in light red. The parts of the network corresponding to inflammation, blood coagulation, and complement system are circled. (**B**) A high-resolution network view of the inflammation system with directionality of the relations (small excerpt depicted). Solid edges represent increase and decrease relations, while dashed edges represent correlations. (**C**) The overlap between the two KGs based on human proteins represented as a Venn diagram. The numbers of nodes that only present proteins are depicted (COVID-19 KG: 595 (red), Heme KG: 106 (blue), overlap between both: 32 (violet)). The two KGs overlapped in the following systems: immune response–inflammation (panel (**D**)), immune response–complement system (panel (**E**)), blood and coagulation system (panel (**F**)), and organ-specific diagnostic markers (panel (**G**)). The hemoglobin level was often decreased in COVID-19 patients [29,59] and is depicted (dashed, red line). ATP: adenosine triphosphate, C3: complement component 3, CRP: C-reactive protein, DPP4: dipeptidyl peptidase 4, HAS2: hyaluronan synthase 2, Hx: hemopexin, IL: interleukin, LDH: lactate dehydrogenase, NKAP: NF-κB-activating protein, PDCD1: programmed cell death protein 1, RIPK3: receptor interacting serine/threonine kinase 3, ROS: reactive oxygen species, and TLR4: Toll-like receptor 4.

In order to investigate the mechanisms linking SARS-CoV-2 and heme, we exploited the KGs generated in our previous work [42,54]. We compiled the two KGs encoded in Biological Expression Language (BEL) using PyBEL [56] directly from their public repositories (i.e., https://github.com/covid19kg and https://github.com/hemekg) (accessed on 11 June 2020) and superimposed their interactions onto a merged network. Given the high degree of expressivity of BEL that enables the representation of multimodal biological information, the KGs were not only enriched with molecular information, but also with interactions from the molecular level to phenotypes and clinical readouts. We leveraged this multimodal information to hypothesize the pathways that connect key molecules associated with SARS-CoV-2 and heme to the phenotypes observed in COVID-19 patients. Since both KGs comprise several thousands of interactions, manually inspecting all relations and evaluating the implication of the crosstalk between COVID-19 and heme was largely infeasible. Accordingly, this analysis primarily focused on the set of nodes present in both KGs. Prior to this crosstalk analysis, we conducted a one-sided Fisher’s exact test [57] to confirm the significance of the overlap between human proteins present in each of the KGs (*p*-value < 0.01). We then manually classified the set of overlapping nodes into four pathways based on their functional role: (i) immune response–inflammation, (ii) immune response–complement system, (iii) blood and coagulation system, and (iv) organ-specific diagnostic markers. Finally, upon superimposing the relations between the overlapping nodes from the Heme and the COVID KGs, we analyzed the signature similarities between each of the above-mentioned pathways. These relations are summarized in Figure 1 and also shown in Supplementary Materials Tables S1–S4 together with their evidence and information on their provenance. In order to validate the hypotheses coming from the KG, we compared the relations emerging from the overlap between the two KGs with experimental data published in the context of COVID-19 [58]. The concordance of the expression patterns in these data sets with each relation is shown in Table S5.

### 2.2. Screening for Potential HBMs in COVID-19-Related Proteins

HeMoQuest (http://131.220.139.55/SeqDHBM/) (accessed on 20 April 2020) [55] was used to identify potential HBMs in proteins of SARS-Cov-2 (i.e., S protein, M protein, E protein, and protein 7a) and human host cells (i.e., ACE2 and TMPRSS2). HBM prediction was refined considering surface accessibility, glycosylation sites, and the involvement in disulfide bonds.

### 2.3. HBM-Peptide Synthesis, Purification and Heme-Binding Analysis

To substantiate the screening for HBMs with the experimental data, the potential motifs found in S protein (3), protein 7a (2), ACE2 (5), and TMPRSS2 (10) were synthesized as nonapeptides as earlier described [60,61]. In brief, automated solid-phase peptide synthesis was performed by standard Fmoc/tBu strategy on Rink amide MBHA resin (loading 0.53 mmol/g). For peptides containing cysteine, the methylated form was used in order to mimic disulfide bond engagement. Preparative reverse-phase high-performance liquid chromatography (RP-HPLC; PU-987, JASCO) was used to purify the crude products. Peptides were characterized by analytical RP-HPLC (LC-20A, Shimadzu) and either LC-ESI-MS (micrOTOF-Q III, Bruker Daltonics; UltiMate 3000 LC, ThermoScientific) or MALDI-TOF-MS (UltrafleXtreme, Bruker Daltonics) (Table S6). Furthermore, amino acid analysis was performed (LC3000, Eppendorf-Biotronik). Using an established setup [61], the heme-binding capacity of the 20 peptides was evaluated by UV-Vis spectroscopy. Heme (0.4–40 µM) was incubated with each peptide (20 µM) for 30 min in HEPES buffer (100 mM, pH 7.0). Afterwards, absorbance spectra (300–600 nm) were measured with a Multiskan GO spectrophotometer (Thermo Fisher Scientific). Difference spectra were generated through spectra subtraction of the single heme and peptide spectra from the heme–peptide complexes. Dissociation constants (K_D_) were calculated by applying the earlier established quadratic equation of Pîrnău and Bogdan (2008) in the program GraphPad Prism 8.0 [61,62].

## 3. Results

COVID-19 progression severely diverged between affected patients with ARDS and other patients, who could even remain asymptomatic. Current research is thus focusing on explaining the reasons for these discrepancies considering the physical conditions and (pre-)existing illnesses of those affected. With regard to a possible interrelation between COVID-19 and heme, several options need to be regarded. Systemic hyperinflammation follows severe COVID-19 infection. It becomes manifest by an increase in the abundance of numerous cytokines (e.g., IL-2, IL-7, IL-6, TNFα) [3], which is indicative of cytokine release syndrome [59] and leads to elevated serum biomarkers in patients (e.g., CRP, lactate dehydrogenase (LDH), D-dimer, ferritin) [2,30,63,64]. Several of these indications, however, were also reported for labile heme occurring in patients with hemolytic disorders [65,66]. In addition, ARDS has been directly correlated with increased levels of labile heme [67]. Thus, the direct interaction of heme with viral surface proteins and host cell proteins should be considered as well. This can be exemplified with the earlier reported interactions of heme with, for example, Zika, chikungunya, and HIV-1 viruses [68,69,70,71]. In the following, we present our results concerning a detailed analysis of the common pathways identified from the Heme KG and COVID-19 KG as well as the potential heme–protein interactions of respective viral and host cell candidates.

### 3.1. Effects of Heme and COVID-19 Intersect at Inflammation

In order to shed light on the crosstalk and common pathways between heme and COVID-19, we investigated the overlap between our two KGs (i.e., heme KG [42] and the COVID-19 KG [54]) (Figure 1A,B). While the Heme KG was generated from the analysis of 46 scientific articles specifically selected to explain inflammatory processes related to labile heme, the COVID-19 KG contains over 150 articles. The difference in the size of these KGs thus explains the disproportionate number of molecules they possess (Figure 1C). Nonetheless, we observed that a significant number of proteins are shared, predominantly in three major systems, namely, blood coagulation, complement, and immune system. Among these 85 shared nodes, there are 45 clinical phenotypes, 35 proteins, four immune system specific cells, and five small molecules. Twenty-seven nodes belong to immune response evoking (pro-)inflammatory pathways, four to the complement system, and 22 to the blood coagulation system (Figure 1). Moreover, we also noticed the presence of seven clinical phenotypes related to organ dysfunction. Further, we individually investigated the four systems to reveal the common relations observed in each of the two KGs (Figure 1, Tables S1–S4).

We compared the directionality of these relations (i.e., up/downregulation) against experimental data published in the context of COVID-19 [58]. We found that the vast majority of the observed dysregulations were concordant with our findings (Table S5). The largest consistency was found in inflammatory pathways (Figure 1B,D) as indicated by a common set of inflammatory—mostly pro-inflammatory—molecules. These molecules are changed, in respect to their levels, due to the expression and/or secretion or their activity as a consequence of both high heme concentrations and COVID-19 infection, mediating inflammatory response. In particular, the pro-inflammatory cytokines TNFα, IL-1β, IL-6, IL-8, and the anti-inflammatory cytokine IL-10 as well as proteins related to TLR4-mediated signaling pathways (i.e., CD14, MyD88, NF-κB, and TLR4) are influenced under both conditions (Figure 1D; Table S1). Within the complement system (Figure 1E; Table S2), one of the main mediators, C3, is activated under hemolytic conditions, which are associated with high heme concentrations thus leading to complement activation [41]. The same was observed in COVID-19 patients [27]. Furthermore, other complement factors, including C5a and C1q, were reported to be activated by heme [41]. So far, an increase in the activation of these proteins was not described for COVID-19. Finally, the number of neutrophils was positively correlated with both heme and COVID-19 infection. However, heme induces neutrophil activation through an ROS-dependent mechanism [39], a pathway that has not yet been discussed in the context of COVID-19. The blood and coagulation system is pronounced by the connecting proteins ferritin and albumin (Figure 1F; Table S3). Both conditions lead to reduced levels of ferritin, a protein involved in iron storage. Same applies for albumin in COVID-19 patients [2,20,21,38]. Moreover, albumin is known as one of the common heme scavengers, neutralizing heme’s toxic effects up to a certain extent [41]. As indicated by the impact on different components of the blood and coagulation system, such as plasminogen or fibrin in the case of COVID-19 and heme, respectively, both conditions can influence hemostasis. With regard to the impact on platelets, a decreased platelet count was observed in COVID-19 patients [28], whereas for heme, an induction of platelet aggregation was described [43]. Finally, a trend towards elevated levels of organ-specific diagnostic markers (i.e., LDH and bilirubin) is shared by both KGs (Figure 1G; Table S4).

### 3.2. Heme-Binding Ability of Proteins of SARS-CoV-2 and Host Cells

**Figure 2:**
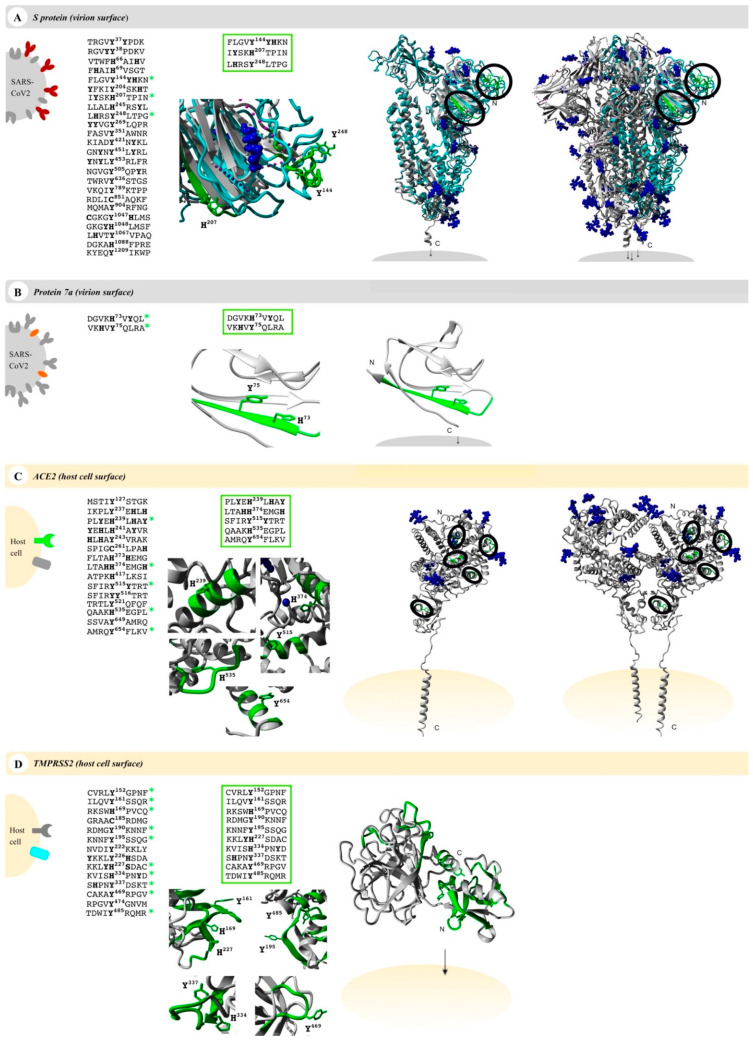
Potential heme-binding proteins on the virus and host cell’s surface. Four COVID-19-related proteins (first column, left), namely, the virus proteins (grey) S protein (red, panel (**A**)) and protein 7a (orange, panel (**B**)) as well as the host cell proteins (yellow) ACE2 (green, panel (**C**)) and TMPRSS2 (turquoise, panel (**D**)), represent potential heme-binding proteins. All motifs predicted by HeMoQuest [55] are shown excluding those with modifications (glycosylation, disulfide bonds) or located in intracellular or virion domains. A refined analysis considering the surface accessibility of the motifs resulted in the following number of motifs: 3 (S protein), 2 (protein 7a), 5 (ACE2), and 10 (TMPRSS2) (third column). The motifs are highlighted in a zoom-in below (green; third column) as well as in the available monomer (fourth column) and oligomer (fifth column) structures, if applicable (S protein, homology model from C-I-TASSER [76]; protein 7a, PDB: 6W37; ACE2, PDB: 6M18; TMPRSS2, Swiss-model: O15393). Within the oligomers, the motifs were only depicted in one of the monomers (green). Since some surface-exposed motifs in the S protein were not covered by the available EM structure (PDB: 6VXX), motifs were highlighted in the monomer (turquoise), which was then superimposed with the trimer (PDB: 6VXX). Blue: glycosylation sites and ions (if applicable).

Numerous interesting target proteins of the virus’ surface and the host cell’s outer membrane were linked with the pathological effects of SARS-CoV-2 including E protein, S protein, M protein, and protein 7a as well as the human proteins ACE2 and TMPRSS2. All proteins contain at least an extracellular, surface-exposed part and are thus accessible for interaction with heme [72,73,74,75]. This led us to examine these proteins for potential heme-binding sites. It is known that regulatory heme binding occurs on surface-exposed HBMs. We identified potential HBMs in all target proteins using the recently published machine-learning web application HeMoQuest [55], which predicts HBMs from primary structure and was trained on a large array of heme-binding peptides. Screening of the amino acid sequences of S protein, protein 7a, ACE2, and TMPRSS2 resulted in 50, 6, 21, and 32 potential HBMs, respectively. M protein and E protein were dismissed as candidates, since no suitable HBMs were found. HBMs, which are part of the transmembrane or intravirion/intracellular domains, were removed from the selection, too. In addition, we excluded motifs in which the central coordinating residue was involved in disulfide bonds or where adjacent residues were glycosylated in the protein. After this refinement of the hits, we identified 24 motifs in S protein, two in protein 7a, 15 in ACE2, and 14 in TMPRSS2 (Figure 2).

**Figure 3:**
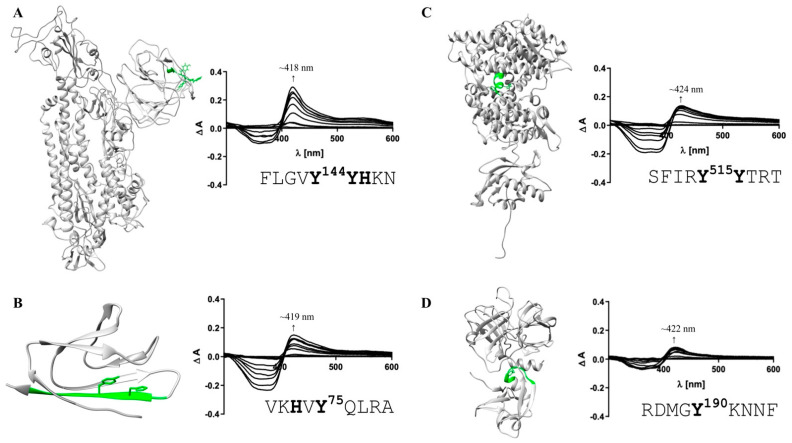
Heme-binding capacity of the most promising HBMs in SARS-CoV-2 and host cell proteins. Heme (0.4–40 µM)-binding properties of the motif-derived peptides (10 µM) were investigated by UV-Vis spectroscopy. The locations of the most promising HBMs within the proteins’ structures (extracellular part; left) and the difference spectra (right) are depicted. (**A**) With a K_D_ of 0.96 ± 0.47 µM (right), FLGV**Y**^144^**YH**KN (green) was the HBM with the highest heme-binding affinity in the S protein. (**B**) For protein 7a (PDB: 6W37), VK**H**V**Y**^75^QLRA was confirmed as a HBM (K_D_ determination not possible). (**C**) Within human ACE2, the motif SFIR**Y**^515^**Y**TRT (green) exhibited the highest heme-binding affinity (K_D_ = 0.60 ± 0.33 µM). (**D**) For TMPRSS2, one HBM with high heme-binding affinity in the SRCR domain was identified (RDMGY^190^KNNF; K_D_ = 0.94 ± 0.38 µM). HBMs that emerged only as moderate heme binders are not shown (Figure S1).

These motifs were then manually screened for surface accessibility using the available protein X-ray or EM structures (Figure 2). Consequently, three motifs for S protein, two motifs for protein 7a, five motifs for ACE2, and ten motifs for TMPRSS2 remained (Figure 2). These were synthesized as nonapeptides for studying their heme-binding capacity, which enabled experimental classification and validation of predicted HBMs. The potential HBMs in S protein were all located in the N-terminal domain of the S1 subunit (Figure 2A) [77,78]. The first occurring sequence, FLGV**Y**^144^**YH**KN, is the most promising HBM, and it is based on a YYH motif and further equipped with phenylalanine at position 4, two additional hydrophobic amino acids (Val, Leu), and a net charge of +2, all beneficial for heme binding [79]. This prediction was validated by UV-Vis binding studies that revealed a high heme-binding affinity (K_D_ = 0.96 ± 0.47 µM; Figure 3A) for this motif.

According to the best fit, a stoichiometry of 1:2 peptide to heme was determined. While at the peptide level, heme binding at two sites is conceivable, at the protein level heme binding to two coordination sites within the motif is rather unlikely due to the steric hindrance by adjacent residues of the protein. The other two potential HBMs, I**Y**SK**H**^207^TPIN and L**H**RS**Y**^248^LTPG, contain a Y/H-based motif with two spacers between the potential coordinating residues (e.g., YXXH) that were shown to be less favorable for heme binding [79]. These motifs showed only a moderate (K_D_ = 7.87 ± 0.64 µM) and very low heme-binding affinity (K_D_ = 26.09 ± 4.75 µM; Figure S1), respectively, rendering both unsuitable for strong heme binding to the S protein. In protein 7a, only two overlapping motifs were predicted, which was not surprising due to the small size of 121 amino acids (Figure 2B). Both DGVK**H**^73^V**Y**QL and VK**H**V**Y**^75^QLRA possess an HXY motif [79] and three hydrophobic residues, rendering it a moderate heme binder and, in turn, protein 7a as a less interesting candidate for interaction with heme. While motif DGVK**H**^73^V**Y**QL did not bind heme at all in vitro, heme binding was observed for the second motif (i.e., VK**H**V**Y**^75^QLRA). These motifs are only shifted by two amino acids and, thus, overlaying. However, due to the N-terminal asparagine acid residue the first motif (i.e., DGVK**H**^73^V**Y**QL) has a more acidic character that can abolish binding of heme. For the second motif (i.e., VK**H**V**Y**^75^QLRA), no binding affinity could be determined (n. sat.), but binding was observed by a shift of the heme Soret band to ~419 nm (Figure 3B). Heme binding to protein 7a might thus be possible but is not very pronounced. The analysis of ACE2 revealed five potential HBMs in total, two of which represent promising H/Y motifs (Figure 2C). The most interesting HBM was LTA**HH**^374^EMG**H**, comprising an HXXXH motif, which was recently shown to exhibit high heme-binding affinity [79]. The central H374 is immediately adjacent to the site that is essential for cleavage by ADAM17 and part of the zinc(II) ion binding site of ACE2 [80,81,82]. However, in vitro, only a very low heme-binding affinity was observed, which excludes this motif as a suitable HBM at the protein level. Although the occurrence of three histidines may be favorable for heme binding, the presence of E^375^ is detrimental. Since it is the zinc(II) ion binding site within ACE2, the present zinc(II) ion might abolish heme binding as well. The second interesting motif was PL**Y**E**H**^239^L**H**A**Y**, since it contains the efficient HXH motif [79] with further advantageous aromatic tyrosines (Y^237^, Y^243^) and hydrophobic leucines (L^236^, L^240^). Again, affinity is limited due to the acidic E^238^, which resulted in a moderate heme-binding affinity (K_D_ = 4.04 ± 1.20 µM). The third motif, SFIR**Y**^515^**Y**TRT, has a YY motif. Although such motifs were shown to be less favorable with respect to affinity [79], this motif displays two basic residues that support heme binding to the motif with high affinity (K_D_ ~0.60 µM; Figure 3C). The remaining two motifs, QAAK**H**^535^EGPL and AMRQ**Y**^654^FLKV, are less promising because they only contain one coordinating amino acid. However, while QAAK**H**^535^EGPL could be excluded as a potential HBM due to the lack of heme binding in vitro, moderate heme-binding affinity to AMRQ**Y**^654^FLKV (K_D_ = 5.01 ± 0.78 µM; Figure S1; Table S6) was demonstrated. As such, ACE2 possesses one HBM with high heme-binding affinity and two HBMs with moderate heme-binding affinity. The largest number of motifs (10 in total) was identified in TMPRSS2 (Figure 2D). Seven of these motifs contain only one coordinating amino acid including those with an additional but hindered cysteine residue. However, one of these, RDMG**Y**KNNF, exhibited a high heme-binding affinity (K_D_ = 0.94 ± 0.38 µM), suggesting it as the most promising motif in TMPRSS2 (Figure 3D). The others either did not show heme binding, possessed only a very low affinity towards heme, or a K_D_ value could not be determined (Figure S1; Table S6). Two further overlapping motifs (i.e., KVIS**H**^334^PN**Y**D and S**H**PN**Y**^337^DSKT) were found in the protease domain of TMPRSS2 (Figure 2D). Such motifs (HXXY) have been demonstrated to be less favorable, as aforementioned, for two motifs from the S protein. In vitro, KVIS**H**^334^PN**Y**D bound heme with moderate affinity, whereas S**H**PN**Y**^337^DSKT did not bind. Interestingly, the motif KVIS**H**^334^PN**Y**D represents a potential heme-binding sites directly in the catalytic protease domain of TMPRSS2. Within the scavenger receptor cysteine-rich (SRCR) domain of the enzyme [72], the interesting motif KKL**YH**^227^SDAC was found. It features a YH motif of intermediate heme-binding affinity on the peptide level, however, of markedly improved affinity on the protein level as earlier demonstrated for IL-36α and activated protein C (APC) [79,83,84]. In TMPRSS2, it has a high positive net charge and a hydrophobic leucine, which likely leads to high heme-binding affinity. With UV-Vis binding studies, binding to the motif was observed; however, the heme-binding affinity could not be determined due to the missing saturation (Figure S1; Table S6).

## 4. Discussion

**Figure 4:**
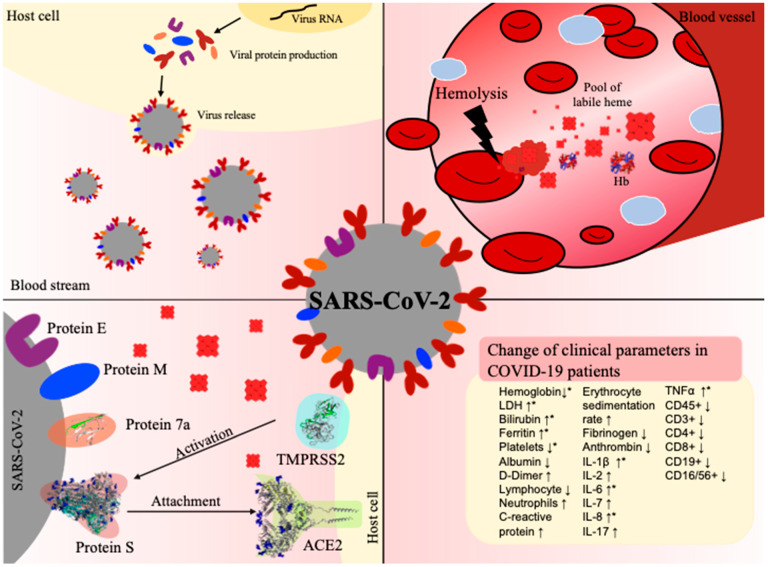
COVID-19 infection and hemolysis show common changes in clinical parameters. Top left: Virus (grey) release after conquering the host cell (yellow) and taking over its protein synthesis machinery. Top right: In the case of hemolysis, erythrocyte lysis occurs in a blood vessel, leading to degradation of hemoglobin (Hb) and, thus, to an excess of labile heme. Bottom left: Interaction between the virus and host cell before cell entry, involving in particular, the viral S protein and protein 7a as well as the human proteins TMPRSS2 and ACE2. Bottom right: Prominent changes in clinical parameters in patients suffering from COVID-19 infection (↑increase, ↓decrease). The terms depicted by an asterisk (*) have been reported in both hemolysis and COVID-19 infection [20,60]. PDBs: 1GZX (hemoglobin), 6VXX (S protein), 6M18 (ACE2), 6W37 (protein 7a).

SARS-CoV-2 and its associated disease, COVID-19, still keeps the world in suspense. The most severely affected patients suffer from pneumonia, acute respiratory distress syndrome, and death [2,3,4]. While COVID-19 patients often exhibit high levels of proinflammatory markers as well as an activation of the complement and the coagulation system, hemoglobin and albumin levels have been reported to be remarkably low [3,20,27,29]. In contrast, there is evidence for higher levels of hemopexin and haptoglobin in COVID-19 patients [29]. These affected clinical parameters have generated a debate about the role of heme in the context of COVID-19 that has not been conclusively explained to date [85,86]. With this work, we intend to provide deeper insights into a potential correlation between SARS-CoV-2 infection, COVID-19, and the effects of heme. Such a connection would be in line with studies that already described the impact of heme in the context of different viruses [68,69,71]. Lecerf et al. reported on the interaction of heme with antibodies (Abs) resulting in the induction of new antigen-binding specificity and acquisition of binding polyreactivity to gp120 HIV-1 in 24% of the antibodies from different B cell subpopulations of seronegative individuals [68]. The transient interaction of heme with a fraction of circulating Abs that might change their antigen-binding repertoire was suggested as another possible regulatory function of heme [68]. In addition, the novel antigen specificities of these circulating Abs was proposed to be recruited only in cases of certain pathological conditions that might depend on extracellular heme such as those occurring in hemolytic disorders [68]. A similar report by Gupta et al. revealed the heme-mediated induction of monoclonal immunoglobulin G1 antibodies that acquired high-affinity reactivity towards antigen domain III of the Japanese encephalitis virus (JEV) E glycoprotein and exhibited neutralizing activity against dominant JEV genotypes [69]. In both cases, heme was found to confer novel binding specificities to the respective Abs without changing the binding to their cognate antigen and, as a consequence of the contact with heme, the anti-inflammatory potential of these Abs was substantially increased [69]. Finally, the inactivation of different arthropod-borne viruses, such as dengue, yellow fever, Zika, and chikungunya, by porphyrin treatment has been described to occur through targeting of the viral envelope and, thus, the early steps of viral infection [70,71]. All together, these studies advocate for studying the impact of heme on coronavirus-infected patients (Figure 4).

We herein investigated the possibility of a direct interaction between heme with SARS-CoV-2 surface proteins and their human counterparts ACE2 and TMPRSS2. Our analysis revealed that heme binding conferred by HBMs is possible for all of these proteins. In the case of S protein, the location of the most promising HBM correlated with the important S1 subunit of the protein, which is responsible for binding to ACE2 (Figure 3A). This potential heme interaction would be of a transient nature, as has been observed for other heme-binding proteins such as IL-36α and APC [83,84]. In order to further proof these predictions, UV-Vis binding studies with motif-derived peptides and heme were conducted. In the past, heme-binding studies on the level of protein-derived peptides as a tool for the evaluation of motifs derived from proteins were successful in characterizing heme binding, as can be exemplified with IL-36α and APC [83,84]. In addition, heme binding to the S1 subunit of the S protein was recently analyzed with surface plasmon resonance spectroscopy, which revealed a binding affinity of ~7 nM [87]. The study focused on bilirubin binding to the S protein, yet lacked information about heme-binding sites, stoichiometry, and the functional consequences of heme binding. However, the observation of direct binding to the S1 subunit clearly supports our findings, since all of the herein identified and validated motifs were found within the S1 subunit (Figure 3A and Figure S1; Table S6). For protein 7a, the central heme-binding motif, VKHVY^75^QLRA, was confirmed (Figure 3B and Figure S1). The function of this protein is not yet fully understood. However, a contribution to the COVID-19 pathogenesis through induction of apoptotic pathways is expected [88] and, thus, could be affected by heme binding as well. From the pool of potential HBMs that were predicted for ACE2, three motifs were identified as suitable motifs with moderate to high heme-binding affinity (Figure 3C and Figure S1; Table S6). All of them are part of the catalytically active zinc metallopeptidase domain of the enzyme. Transient heme binding might thus lead to a change in the catalytic activity of the enzyme, as earlier observed, for example, with APC [84] or hemolysin C [89]. The same applies for TMPRSS2. One HBM with high heme-binding affinity is part of the SRCR domain and two HBMs with moderate heme-binding affinity were found within the peptidase domain. Future binding and functional studies on the protein level for COVID-19-infection-related proteins are required to enable a complete assessment of the suggested interactions. Thereby, the correct nature of these proteins, including glycosylation, folding, and multimerization, should be considered, which is currently restricted or non-existent with regard to purchasable proteins.

Apart from investigating the direct impact of heme on proteins at the interface of the virus–host cell interaction, we also explored similarities between relevant pathways characterizing the respective pathologies, i.e., labile heme occurrence in hemolytic conditions and COVID-19 disease progression (Figure 4). Interestingly, we found several commonalities in the literature: both hemolytic conditions and COVID-19 have been found to trigger inflammatory pathways. COVID-19 patients often develop respiratory distress syndrome, which is accompanied by a cytokine storm and, thus, an activation of the immune system [3]. Clinically, this is manifested by an increase in the levels of a wide range of cytokines, including TNFα, IL-1β, IL-6, and IL-8 [3], and the activation of the complement system (e.g., C3) [27,90]. The hemoglobin level is often decreased in COVID-19 patients [29,59], but the underlying molecular mechanism of this phenomenon is not yet identified [85,91,92]. However, the lower levels seem to correlate with increased levels of the iron-storage protein ferritin. Ferritin is also upregulated during hemolytic diseases as a consequence of hemoglobin degradation and the associated increase in oxidative stress, for example, induced by heme [93]. Hemolytic disorders, such as malaria and ischemia reperfusion, are associated with an excess of labile heme and are, as in COVID-19 infection, often accompanied by inflammatory events [46,66]. Therefore, similar clinical parameters are observed under these conditions [66]. Moreover, several studies have reported that heme directly binds or induces TNFα, IL-1β, and IL-8, and triggers numerous inflammatory pathways (e.g., NF-κB signaling) [39,42]. Taken together, these clinical observations suggest a correlation between both processes, which we aimed to analyze by superimposing the two KGs of both pathophysiologies [42,54]. We overlayed the Heme KG with the COVID-19 KG to identify similarities, as successfully shown in the earlier reported approach for type II diabetes and Alzheimer’s disease [50,51,53]. Indeed, the results of the knowledge-driven analysis revealed a core of similar shared molecular patterns. The majority of these were related to three major systems: inflammation, complement, and coagulation. As expected, inflammation was the most emphasized and shared system, suggesting several processes that are commonly mediated by both heme and in COVID-19 pathogenesis. The TLR4 signaling pathway was previously shown to play an important role in heme-mediated inflammatory processes [42,49]. Interestingly, this pathway with its components, TLR4, MyD88, and NF-κB, was pronounced in the overlay of the heme KG and COVID-19 KG. The TLR4 pathway belongs to the innate immune system and, thus, results in the production of several proinflammatory cytokines such as TNFα, IL-1β, and IL-6 [42]. TNFα and IL-1β can further stimulate the release of inflammatory mediators such as IL-8. Exactly the same proteins have emerged as common key molecules in our analysis. Clinical observations revealed their upregulation in COVID-19 patients as well as during hemolytic events [3,39,42], which highlights even more the TLR4 signaling pathway in both situations. Interestingly, TNFα and IL-1β were reported to be capable of regulating platelet aggregation. This supports the common link of both pathologies to blood coagulation [94]. Blood parameters, such as hemoglobin and albumin levels, may allow for a direct correlation between COVID-19 progression and heme availability, since they are inevitably connected to the processing of hemoglobin and heme, respectively, under hemolytic conditions [41,46]. In the current state of research, there is no explanation for the decreased levels of hemoglobin in COVID-19 patients. It might be conceivable that it is due to the rapid turnover of red blood cells, which would lead to a degradation of hemoglobin and, in turn, to an increase in heme. SARS-CoV-2 infections were recently associated with anemia, hemolytic, and/or hemorrhagic conditions [95,96,97,98,99]. Moreover, increased heme levels (~20 µM), comparable to hemolytic conditions [100,101], were detected in COVID-19 patients [102], which further supports the importance of the results gained within this study and with respect to the need for developing tailor-made diagnosis and therapeutic strategies for these patients.

## 5. Conclusions

In summary, commonalities between COVID-19 pathophysiology and heme-driven complications under hemolytic conditions were investigated using a computational approach and a possible link supported by in vitro studies of select heme–ligand interactions derived from COVID-19-related proteins.

First, a detailed analysis of common pathways was conducted through the superimposition of two knowledge graphs, i.e., the “Heme KG” [42] and the “COVID-19 KG” [54] that encompassed information of heme-driven and COVID-19 pathophysiologies on a molecular basis. In support of the obviously common clinical parameters, several biomarkers emerged that belonged to proinflammatory pathways (cytokines, such as TNFα), the complement system (e.g., C3), the blood and coagulation system (e.g., ferritin, platelets) as well as organ-specific markers (e.g., LDH).

In a second approach, four proteins that participate in the entry of SARS-CoV-2 into host cells (i.e., the two viral proteins S protein and protein 7a as well as the two human host cell proteins ACE2 and TMPRSS2) were predicted as potential heme-binding proteins (out of six analyzed COVID-19-related proteins). The heme-binding potential of these proteins was evaluated with the help of the web application HeMoQuest [55] and manual refinement, which revealed two potential HBMs on the surface of protein 7a, three for S protein, five for ACE2, and 10 motifs for TMPRSS2.

Finally, heme binding to these motifs was further analyzed in vitro using respective peptide models (HBMs). UV-Vis spectroscopy allowed for the evaluation of the heme-binding capacity and affinity of the heme association with the individual motifs. The presence of at least one highly favorable HBM for each of the four proteins was confirmed.

In conclusion, the results of this study draw attention to a relationship that is plausible based on the current characterization of COVID-19 pathogenesis by clinical parameters. A correlation between the symptoms of COVID-19 infection and the consequences of excess heme does not necessarily have to be relevant for each patient, but in specific cases it may correlate or even cause a more severe progression of the disease due to the fact of pre-existing hemolytic conditions or hemolysis-provoking events.

## Data Availability

The data sets supporting the conclusions of this article are included within the article and its supplementary materials. The herein used knowledge graphs and the web application HeMoQuest are publicly accessible at https://github.com/covid19kg (accessed on 11 June 2020), https://github.com/hemekg (accessed on 11 June 2020) and http://131.220.139.55/SeqDHBM/ (accessed on 20 April 2020), respectively.

## Supplementary Materials

The following are available online at https://www.mdpi.com/article/10.3390/biom11050644/s1, Figure S1: Subordinate motifs in SARS-CoV-2 proteins and human host cell proteins, Table S1: Original evidence for the depicted connections for “immune response–inflammation”, Table S2: Original evidence for the depicted connections for “immune response–complement system”, Table S3: Original evidence for the depicted connections for “blood and coagulation system”, Table S4: Original evidence for the depicted connections for “organ-specific diagnostic markers”, Table S5: Concordance between common interactions and experimental data, Table S6: Characterization of potential heme-binding motifs within SARS-CoV-2 proteins and human host cell proteins.

## References

[1] Cucinotta D., Vanelli M. (2020). WHO declares COVID-19 a pandemic. Acta Biomed..

[2] Zhou F., Yu T., Du R., Fan G., Liu Y., Liu Z., Xiang J., Wang Y., Song B., Gu X. (2020). Clinical course and risk factors for mortality of adult inpatients with COVID-19 in Wuhan, China: A retrospective cohort study. Lancet.

[3] Ye Q., Wang B., Mao J. (2020). The pathogenesis and treatment of the Cytokine Storm in COVID-19. J. Infect..

[4] Ragab D., Salah Eldin H., Taeimah M., Khattab R., Salem R. (2020). The COVID-19 cytokine storm: What we know so far. Front. Immunol..

[5] Hanff T.C., Harhay M.O., Brown T.S., Cohen J.B., Mohareb A.M. (2020). Is there an association between COVID-19 mortality and the renin-angiotensin system?—A call for epidemiologic investigations. Clin. Infect. Dis..

[6] Mascolo A., Scavone C., Rafaniello C., Ferrajolo C., Racagni G., Berrino L., Paolisso G., Rossi F., Capuano A. (2020). Renin-angiotensin system and coronavirus disease 2019: A narrative review. Front. Cardiovasc. Med..

[7] Yi C., Sun X., Ye J., Ding L., Liu M., Yang Z., Lu X., Zhang Y., Ma L., Gu W. (2020). Key residues of the receptor binding motif in the spike protein of SARS-CoV-2 that interact with ACE2 and neutralizing antibodies. Cell. Mol. Immunol..

[8] Hoffmann M., Kleine-Weber H., Schroeder S., Krüger N., Herrler T., Erichsen S., Schiergens T.S., Herrler G., Wu N.-H., Nitsche A. (2020). SARS-CoV-2 cell entry depends on ACE2 and TMPRSS2 and is blocked by a clinically proven protease inhibitor. Cell.

[9] Ali A., Vijayan R. (2020). Dynamics of the ACE2–SARS-CoV-2/SARS-CoV spike protein interface reveal unique mechanisms. Sci. Rep..

[10] Xue X., Shi J., Xu H., Qin Y., Yang Z., Feng S., Liu D., Jian L., Hua L., Wang Y. (2021). Dynamics of binding ability prediction between spike protein and human ACE2 reveals the adaptive strategy of SARS-CoV-2 in humans. Sci. Rep..

[11] Ren X. (2006). Analysis of ACE2 in polarized epithelial cells: Surface expression and function as receptor for severe acute respiratory syndrome-associated coronavirus. J. Gen. Virol..

[12] Wu K., Peng G., Wilken M., Geraghty R.J., Li F. (2012). Mechanisms of host receptor adaptation by severe acute respiratory syndrome coronavirus. J. Biol. Chem..

[13] Zhai X., Sun J., Yan Z., Zhang J., Zhao J., Zhao Z., Gao Q., He W.-T., Veit M., Su S. (2020). Comparison of severe acute respiratory syndrome coronavirus 2 spike protein binding to ACE2 receptors from human, pets, farm animals, and putative intermediate hosts. J. Virol..

[14] Bianchi M., Benvenuto D., Giovanetti M., Angeletti S., Ciccozzi M., Pascarella S. (2020). Sars-CoV-2 envelope and membrane proteins: Structural differences linked to virus characteristics?. Biomed. Res. Int..

[15] Boson B., Legros V., Zhou B., Siret E., Mathieu C., Cosset F.-L., Lavillette D., Denolly S. (2021). The SARS-CoV-2 envelope and membrane proteins modulate maturation and retention of the spike protein, allowing assembly of virus-like particles. J. Biol. Chem..

[16] Fielding B.C., Gunalan V., Tan T.H.P., Chou C.-F., Shen S., Khan S., Lim S.G., Hong W., Tan Y.-J. (2006). Severe acute respiratory syndrome coronavirus protein 7a interacts with hSGT. Biochem. Biophys. Res. Commun..

[17] Kwon S., Kim E., Jung Y.S., Jang J.S., Cho N. (2020). Post-donation COVID-19 identification in blood donors. Vox Sang..

[18] Rosenthal S.H., Kagan R.M., Gerasimova A., Anderson B., Grover D., Hua M., Liu Y., Owen R., Lacbawan F. (2020). Identification of eight SARS-CoV-2 ORF7a deletion variants in 2726 clinical specimens. bioRxiv.

[19] Zhou Z., Huang C., Zhou Z., Huang Z., Su L., Kang S., Chen X., Chen Q., He S., Rong X. (2020). Structural insight reveals SARS-CoV-2 Orf7a as an immunomodulating factor for human CD14+ monocytes. SSRN Electron. J..

[20] Chen N., Zhou M., Dong X., Qu J., Gong F., Han Y., Qiu Y., Wang J., Liu Y., Wei Y. (2020). Epidemiological and clinical characteristics of 99 cases of 2019 novel coronavirus pneumonia in Wuhan, China: A descriptive study. Lancet.

[21] Guan W., Ni Z., Hu Y., Liang W., Ou C., He J., Liu L., Shan H., Lei C., Hui D.S.C. (2020). Clinical characteristics of coronavirus disease 2019 in China. N. Engl. J. Med..

[22] Han H., Yang L., Liu R., Liu F., Wu K., Li J., Liu X., Zhu C. (2020). Prominent changes in blood coagulation of patients with SARS-CoV-2 infection. Clin. Chem. Lab. Med..

[23] Latz C.A., DeCarlo C., Boitano L., Png C.Y.M., Patell R., Conrad M.F., Eagleton M., Dua A. (2020). Blood type and outcomes in patients with COVID-19. Ann. Hematol..

[24] Li J., Wang X., Chen J., Cai Y., Deng A., Yang M. (2020). Association between ABO blood groups and risk of SARS-CoV-2 pneumonia. Br. J. Haematol..

[25] Wu B.-B., Gu D.-Z., Yu J.-N., Yang J., Shen W.-Q. (2020). Association between ABO blood groups and COVID-19 infection, severity and demise: A systematic review and meta-analysis. Infect. Genet. Evol..

[26] Zhao J., Yang Y., Huang H., Li D., Gu D., Lu X., Zhang Z., Liu L., Liu T., Liu Y. (2020). Relationship between the ABO blood group and the coronavirus disease 2019 (COVID-19) susceptibility. Clin. Infect. Dis..

[27] Risitano A.M., Mastellos D.C., Huber-Lang M., Yancopoulou D., Garlanda C., Ciceri F., Lambris J.D. (2020). Complement as a target in COVID-19?. Nat. Rev. Immunol..

[28] Wang R., Pan M., Zhang X., Han M., Fan X., Zhao F., Miao M., Xu J., Guan M., Deng X. (2020). Epidemiological and clinical features of 125 Hospitalized Patients with COVID-19 in Fuyang, Anhui, China. Int. J. Infect. Dis..

[29] Whetton A.D., Preston G.W., Abubeker S., Geifman N. (2020). Proteomics and informatics for understanding phases and identifying biomarkers in COVID-19 disease. J. Proteome Res..

[30] Zhang X., Cai H., Hu J., Lian J., Gu J., Zhang S., Ye C., Lu Y., Jin C., Yu G. (2020). Epidemiological, clinical characteristics of cases of SARS-CoV-2 infection with abnormal imaging findings. Int. J. Infect. Dis..

[31] Tang N., Li D., Wang X., Sun Z. (2020). Abnormal coagulation parameters are associated with poor prognosis in patients with novel coronavirus pneumonia. J. Thromb. Haemost..

[32] Ji H.-L., Zhao R., Matalon S., Matthay M.A. (2020). Elevated plasmin(ogen) as a common risk factor for COVID-19 susceptibility. Physiol. Rev..

[33] Grobler C., Maphumulo S.C., Grobbelaar L.M., Bredenkamp J.C., Laubscher G.J., Lourens P.J., Steenkamp J., Kell D.B., Pretorius E. (2020). Covid-19: The rollercoaster of fibrin(ogen), D-Dimer, von Willebrand factor, P-selectin and their interactions with endothelial cells, platelets and erythrocytes. Int. J. Mol. Sci..

[34] Giannis D., Ziogas I.A., Gianni P. (2020). Coagulation disorders in coronavirus infected patients: COVID-19, SARS-CoV-1, MERS-CoV and lessons from the past. J. Clin. Virol..

[35] Manne B.K., Denorme F., Middleton E.A., Portier I., Rowley J.W., Stubben C., Petrey A.C., Tolley N.D., Guo L., Cody M. (2020). Platelet gene expression and function in patients with COVID-19. Blood.

[36] Leppkes M., Knopf J., Naschberger E., Lindemann A., Singh J., Herrmann I., Stürzl M., Staats L., Mahajan A., Schauer C. (2020). Vascular occlusion by neutrophil extracellular traps in COVID-19. EBioMedicine.

[37] Zuo Y., Warnock M., Harbaugh A., Yalavarthi S., Gockman K., Zuo M., Madison J.A., Knight J.S., Kanthi Y., Lawrence D.A. (2021). Plasma tissue plasminogen activator and plasminogen activator inhibitor-1 in hospitalized COVID-19 patients. Sci. Rep..

[38] Yang X., Yu Y., Xu J., Shu H., Xia J., Liu H., Wu Y., Zhang L., Yu Z., Fang M. (2020). Clinical course and outcomes of critically ill patients with SARS-CoV-2 pneumonia in Wuhan, China: A single-centered, retrospective, observational study. Lancet Respir. Med..

[39] Dutra F.F., Bozza M.T. (2014). Heme on innate immunity and inflammation. Front. Pharmacol..

[40] Kühl T., Imhof D. (2014). Regulatory Fe (II/III) heme: The reconstruction of a molecule’s biography. ChemBioChem.

[41] Roumenina L.T., Rayes J., Lacroix-Desmazes S., Dimitrov J.D. (2016). Heme: Modulator of plasma systems in hemolytic diseases. Trends Mol. Med..

[42] Humayun F., Domingo-Fernández D., Paul George A.A., Hopp M.-T., Syllwasschy B.F., Detzel M.S., Hoyt C.T., Hofmann-Apitius M., Imhof D. (2020). A computational approach for mapping heme biology in the context of hemolytic disorders. Front. Bioeng. Biotechnol..

[43] Hopp M.-T., Imhof D. (2021). Linking labile heme with thrombosis. J. Clin. Med..

[44] Ascenzi P., Bocedi A., Visca P., Altruda F., Tolosano E., Beringhelli T., Fasano M. (2005). Hemoglobin and heme scavenging. IUBMB Life.

[45] Gouveia Z., Carlos A.R., Yuan X., Aires-da-Silva F., Stocker R., Maghzal G.J., Leal S.S., Gomes C.M., Todorovic S., Iranzo O. (2017). Characterization of plasma labile heme in hemolytic conditions. FEBS J..

[46] Chiabrando D., Vinchi F., Fiorito V., Mercurio S., Tolosano E. (2014). Heme in pathophysiology: A matter of scavenging, metabolism and trafficking across cell membranes. Front. Pharmacol..

[47] Martins R., Maier J., Gorki A.-D., Huber K.V.M., Sharif O., Starkl P., Saluzzo S., Quattrone F., Gawish R., Lakovits K. (2016). Heme drives hemolysis-induced susceptibility to infection via disruption of phagocyte functions. Nat. Immunol..

[48] Kupke T., Klare J.P., Brügger B. (2020). Heme binding of transmembrane signaling proteins undergoing regulated intramembrane proteolysis. Commun. Biol..

[49] Janciauskiene S., Vijayan V., Immenschuh S. (2020). TLR4 signaling by heme and the role of heme-binding blood proteins. Front. Immunol..

[50] Kodamullil A.T., Younesi E., Naz M., Bagewadi S., Hofmann-Apitius M. (2015). Computable cause-and-effect models of healthy and Alzheimer’s disease states and their mechanistic differential analysis. Alzheimer’s Dement..

[51] Karki R., Kodamullil A.T., Hofmann-Apitius M. (2017). Comorbidity analysis between Alzheimer’s Disease and type 2 diabetes mellitus (T2DM) based on shared pathways and the role of T2DM drugs. J. Alzheimer’s Dis..

[52] Emon M.A.E.K., Kodamullil A.T., Karki R., Younesi E., Hofmann-Apitius M. (2017). Using Drugs as Molecular Probes: A Computational Chemical Biology Approach in Neurodegenerative Diseases. J. Alzheimer’s Dis..

[53] Karki R., Madan S., Gadiya Y., Domingo-Fernández D., Kodamullil A.T., Hofmann-Apitius M. (2020). Data-driven modeling of knowledge assemblies in understanding comorbidity between type 2 diabetes mellitus and Alzheimer’s Disease. J. Alzheimer’s Dis..

[54] Domingo-Fernández D., Baksi S., Schultz B., Gadiya Y., Karki R., Raschka T., Ebeling C., Hofmann-Apitius M., Kodamullil A.T. (2020). COVID-19 Knowledge Graph: A computable, multi-modal, cause-and-effect knowledge model of COVID-19 pathophysiology. Bioinformatics.

[55] Paul George A.A., Lacerda M., Syllwasschy B.F., Hopp M.-T., Wißbrock A., Imhof D. (2020). HeMoQuest: A webserver for qualitative prediction of transient heme binding to protein motifs. BMC Bioinform..

[56] Hoyt C.T., Konotopez A., Ebeling C. (2018). PyBEL: A computational framework for Biological Expression Language. Bioinformatics.

[57] Fisher R.A. (1992). Statistical methods for research workers. Breakthroughs in Statistics.

[58] Blanco-Melo D., Nilsson-Payant B.E., Liu W.-C., Uhl S., Hoagland D., Møller R., Jordan T.X., Oishi K., Panis M., Sachs D. (2020). Imbalanced host response to SARS-CoV-2 drives development of COVID-19. Cell.

[59] Huang C., Wang Y., Li X., Ren L., Zhao J., Hu Y., Zhang L., Fan G., Xu J., Gu X. (2020). Clinical features of patients infected with 2019 novel coronavirus in Wuhan, China. Lancet.

[60] Kühl T., Sahoo N., Nikolajski M., Schlott B., Heinemann S.H., Imhof D., Kühl T., Heinemann S.H., Schlott B., Imhof D. (2011). Determination of Hemin-Binding Characteristics of Proteins by a Combinatorial Peptide Library Approach. ChemBioChem.

[61] Kühl T., Wißbrock A., Goradia N., Sahoo N., Galler K., Neugebauer U., Popp J., Heinemann S.H., Ohlenschläger O., Imhof D. (2013). Analysis of Fe(III) heme binding to cysteine-containing heme-regulatory motifs in proteins. ACS Chem. Biol..

[62] Pîrnău A., Bogdan M. (2008). Investigation of the interaction between naproxen and human serum albumin. Rom. J. Biophys..

[63] Chen T., Wu D., Chen H., Yan W., Yang D., Chen G., Ma K., Xu D., Yu H., Wang H. (2020). Clinical characteristics of 113 deceased patients with coronavirus disease 2019: Retrospective study. BMJ.

[64] Young B.E., Ong S.W.X., Kalimuddin S., Low J.G., Tan S.Y., Loh J., Ng O.-T., Marimuthu K., Ang L.W., Mak T.M. (2020). Epidemiologic features and clinical course of patients infected with SARS-CoV-2 in Singapore. J. Amer. Med. Assoc..

[65] Litalien C., Proulx F., Mariscalco M.M., Robitaille P., Turgeon J.P., Orrbine E., Rowe P.C., McLaine P.N., Seidman E. (1999). Circulating inflammatory cytokine levels in hemolytic uremic syndrome. Pediatr. Nephrol..

[66] Barcellini W., Fattizzo B. (2015). Clinical applications of hemolytic markers in the differential diagnosis and management of hemolytic anemia. Dis. Markers.

[67] Aggarwal S., Lazrak A., Ahmad I., Yu Z., Bryant A., Mobley J.A., Ford D.A., Matalon S. (2020). Reactive species generated by heme impair alveolar epithelial sodium channel function in acute respiratory distress syndrome. Redox Biol..

[68] Lecerf M., Scheel T., Pashov A.D., Jarossay A., Ohayon D., Planchais C., Mesnage S., Berek C., Kaveri S.V., Lacroix-Desmazes S. (2015). Prevalence and gene characteristics of antibodies with cofactor-induced HIV-1 specificity. J. Biol. Chem..

[69] Gupta N., de Wispelaere M., Lecerf M., Kalia M., Scheel T., Vrati S., Berek C., Kaveri S.V., Desprès P., Lacroix-Desmazes S. (2015). Neutralization of Japanese Encephalitis Virus by heme-induced broadly reactive human monoclonal antibody. Sci. Rep..

[70] Assunção-Miranda I., Cruz-Oliveira C., Neris R.L.S., Figueiredo C.M., Pereira L.P.S., Rodrigues D., Araujo D.F.F., Da Poian A.T., Bozza M.T. (2016). Inactivation of Dengue and Yellow Fever viruses by heme, cobalt-protoporphyrin IX and tin-protoporphyrin IX. J. Appl. Microbiol..

[71] Neris R.L.S., Figueiredo C.M., Higa L.M., Araujo D.F., Carvalho C.A.M., Verçoza B.R.F., Silva M.O.L., Carneiro F.A., Tanuri A., Gomes A.M.O. (2018). Co-protoporphyrin IX and Sn-protoporphyrin IX inactivate Zika, Chikungunya and other arboviruses by targeting the viral envelope. Sci. Rep..

[72] Mendes de Oliveira G., Valle Garay A., Araújo Souza A., Cunha J., Lima B., Fonseca Valadares N., Maria S., Freitas D., Alexandre J., Gonçalves Barbosa R. (2018). Structural characterization and crystallization of human TMPRSS2 protease. Biophys. J..

[73] Mousavizadeh L., Ghasemi S. (2020). Genotype and phenotype of COVID-19: Their roles in pathogenesis. J. Microbiol. Immunol. Infect..

[74] Walls A.C., Park Y.-J., Tortorici M.A., Wall A., McGuire A.T., Veesler D. (2020). Structure, function, and antigenicity of the SARS-CoV-2 spike glycoprotein. Cell.

[75] Hänel K., Willbold D. (2007). SARS-CoV accessory protein 7a directly interacts with human LFA-1. Biol. Chem..

[76] Zhang C., Zheng W., Huang X., Bell E.W., Zhou X., Zhang Y. (2020). Protein structure and sequence reanalysis of 2019-nCoV genome refutes snakes as its intermediate host and the unique similarity between Its spike protein Insertions and HIV-1. J. Proteome Res..

[77] Ou X., Liu Y., Lei X., Li P., Mi D., Ren L., Guo L., Guo R., Chen T., Hu J. (2020). Characterization of spike glycoprotein of SARS-CoV-2 on virus entry and its immune cross-reactivity with SARS-CoV. Nat. Commun..

[78] Huang Y., Yang C., Xu X., Xu W., Liu S. (2020). Structural and functional properties of SARS-CoV-2 spike protein: Potential antivirus drug development for COVID-19. Acta Pharmacol. Sin..

[79] Syllwasschy B.F., Beck M.S., Družeta I., Hopp M.-T., Ramoji A., Neugebauer U., Nozinovic S., Menche D., Willbold D., Ohlenschläger O. (2020). High-affinity binding and catalytic activity of His/Tyr-based sequences: Extending heme-regulatory motifs beyond CP. Biochim. Biophys. Acta Gen. Subj..

[80] Towler P., Staker B., Prasad S.G., Menon S., Tang J., Parsons T., Ryan D., Fisher M., Williams D., Dales N.A. (2004). ACE2 X-ray structures reveal a large hinge-bending motion important for inhibitor binding and catalysis. J. Biol. Chem..

[81] Heurich A., Hofmann-Winkler H., Gierer S., Liepold T., Jahn O., Pohlmann S. (2014). TMPRSS2 and ADAM17 cleave ACE2 differentially and only proteolysis by TMPRSS2 augments entry driven by the severe acute respiratory syndrome coronavirus spike protein. J. Virol..

[82] Lan J., Ge J., Yu J., Shan S., Zhou H., Fan S., Zhang Q., Shi X., Wang Q., Zhang L. (2020). Structure of the SARS-CoV-2 spike receptor-binding domain bound to the ACE2 receptor. Nature.

[83] Wißbrock A., Goradia N.B., Kumar A., Paul George A.A., Kühl T., Bellstedt P., Ramachandran R., Hoffmann P., Galler K., Popp J. (2019). Structural insights into heme binding to IL-36α proinflammatory cytokine. Sci. Rep..

[84] Hopp M.-T., Alhanafi N., Paul George A.A., Hamedani N.S., Biswas A., Oldenburg J., Pötzsch B., Imhof D. (2021). Molecular insights and functional consequences of the interaction of heme with activated protein C. Antioxid. Redox Signal..

[85] Liu W., Li H. (2020). COVID-19: Attacks the 1-beta chain of hemoglobin and captures the porphyrin to inhibit human heme metabolism. ChemRxiv.

[86] Wagener F.A.D.T.G., Pickkers P., Peterson S.J., Immenschuh S., Abraham N.G. (2020). Targeting the heme-heme oxygenase system to prevent severe complications following COVID-19 infections. Antioxidants.

[87] Rosa A., Pye V.E., Graham C., Muir L., Seow J., Ng K.W., Cook N.J., Rees-Spear C., Parker E., dos Santos M.S. (2021). SARS-CoV-2 recruits a haem metabolite to evade antibody immunity. medRxiv.

[88] Michel C.J., Mayer C., Poch O., Thompson J.D. (2020). Characterization of accessory genes in coronavirus genomes. Virol. J..

[89] Peherstorfer S., Brewitz H.H., Paul George A.A., Wißbrock A., Adam J.M., Schmitt L., Imhof D. (2018). Insights into mechanism and functional consequences of heme binding to hemolysin-activating lysine acyltransferase HlyC from Escherichia coli. Biochim. Biophys. Acta Gen. Subj..

[90] Skendros P., Mitsios A., Chrysanthopoulou A., Mastellos D.C., Metallidis S., Rafailidis P., Ntinopoulou M., Sertaridou E., Tsironidou V., Tsigalou C. (2020). Complement and tissue factor–enriched neutrophil extracellular traps are key drivers in COVID-19 immunothrombosis. J. Clin. Investig..

[91] Daniel Y., Hunt B.J., Retter A., Henderson K., Wilson S., Sharpe C.C., Shattock M.J. (2020). Haemoglobin oxygen affinity in patients with severe COVID-19 infection. Br. J. Haematol..

[92] DeMartino A.W., Rose J.J., Amdahl M.B., Dent M.R., Shah F.A., Bain W., McVerry B.J., Kitsios G.D., Tejero J., Gladwin M.T. (2020). No evidence of hemoglobin damage by SARS-CoV-2 infection. Haematologica.

[93] Belcher J.D., Beckman J.D., Balla G., Balla J., Vercellotti G. (2010). Heme degradation and vascular injury. Antioxid. Redox Signal..

[94] Bar J., Zosmer A., Hod M., Elder M.G., Sullivan M.H. (1997). The regulation of platelet aggregation in vitro by interleukin-1beta and tumor necrosis factor-alpha: Changes in pregnancy and in pre-eclampsia. Thromb. Haemost..

[95] Lazarian G., Quinquenel A., Bellal M., Siavellis J., Jacquy C., Re D., Merabet F., Mekinian A., Braun T., Damaj G. (2020). Autoimmune haemolytic anaemia associated with COVID-19 infection. Br. J. Haematol..

[96] Capes A., Bailly S., Hantson P., Gerard L., Laterre P.-F. (2020). COVID-19 infection associated with autoimmune hemolytic anemia. Ann. Hematol..

[97] Conti C.B., Henchi S., Coppeta G.P., Testa S., Grassia R. (2020). Bleeding in COVID-19 severe pneumonia: The other side of abnormal coagulation pattern?. Eur. J. Intern. Med..

[98] Agarwal A., Vishnu V.Y., Vibha D., Bhatia R., Gupta A., Das A., Srivastava M.V.P. (2020). Intracerebral hemorrhage and SARS-CoV-2: Association or causation. Ann. Indian Acad. Neurol..

[99] Sahu K.K., Borogovac A., Cerny J. (2021). COVID-19 related immune hemolysis and thrombocytopenia. J. Med. Virol..

[100] Reiter C.D., Wang X., Tanus-Santos J.E., Hogg N., Cannon R.O., Schechter A.N., Gladwin M.T. (2002). Cell-free hemoglobin limits nitric oxide bioavailability in sickle-cell disease. Nat. Med..

[101] Oh J.-Y., Hamm J., Xu X., Genschmer K., Zhong M., Lebensburger J., Marques M.B., Kerby J.D., Pittet J.-F., Gaggar A. (2016). Absorbance and redox based approaches for measuring free heme and free hemoglobin in biological matrices. Redox. Biol..

[102] Su W.-L., Lin C.-P., Hang H.-C., Wu P.-S., Cheng C.-F., Chao Y.-C. (2021). Desaturation and heme elevation during COVID-19 infection: A potential prognostic factor of heme oxygenase-1. J. Microbiol. Immunol. Infect..

