# Supplementary material for: Linking COVID-19 and Heme-Driven Pathophysiologies: A Combined Computational–Experimental Approach
Source: Biomolecules. 2021 Apr 27;11(5):644. doi: 10.3390/biom11050644 (PMC8147026; doi:10.3390/biom11050644)
Supplement: Supplementary file 1 [file biomolecules-11-00644-s001.zip › biomolecules-1193498-supplementary.pdf]

Supplementary Materials

Supplementary Figures

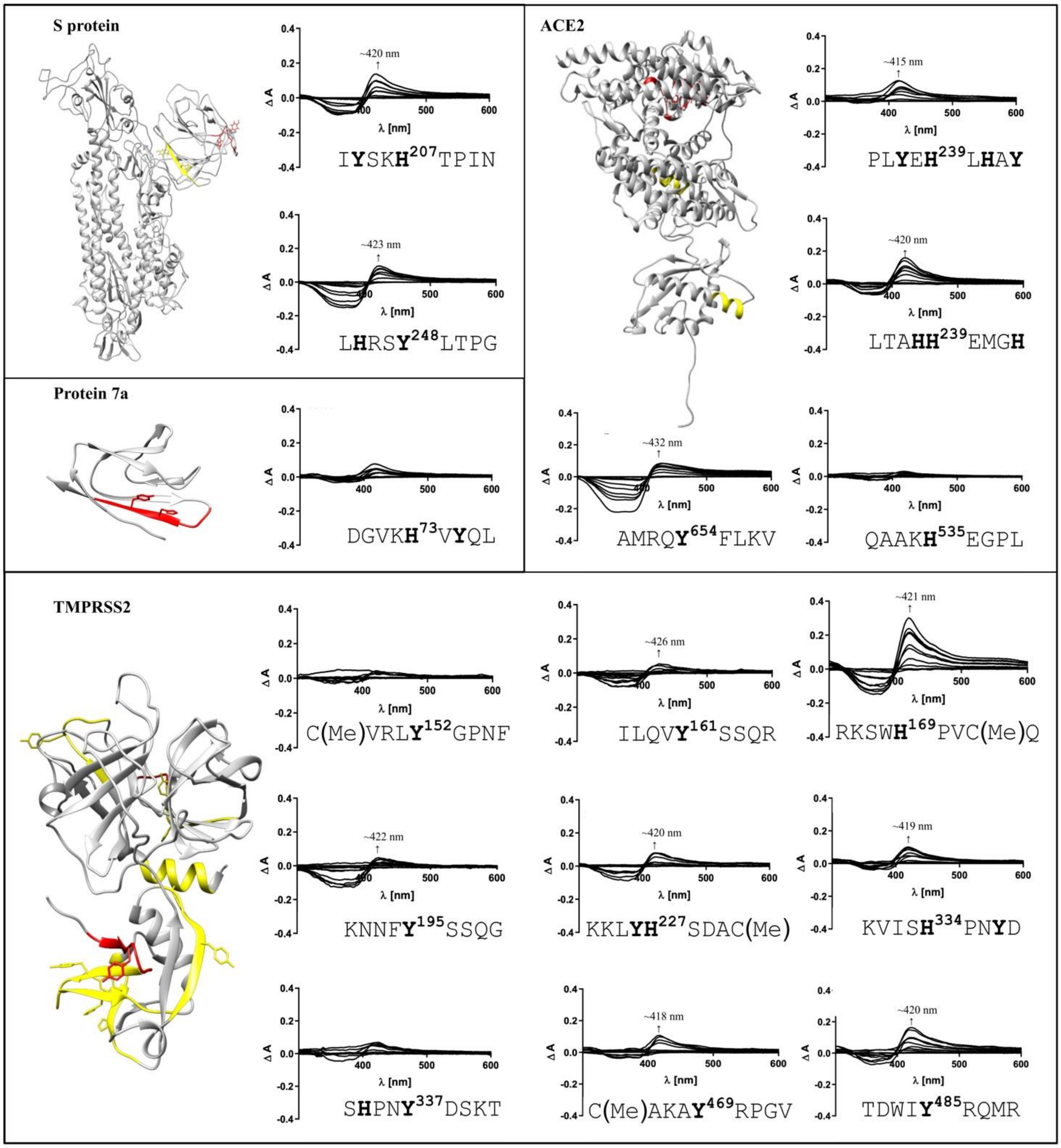

Figure S1. Subordinate motifs in SARS-CoV-2 proteins (i.e., S protein and protein 7a) and human host cell proteins (i.e., ACE2 and TMPRSS2). The location of non-binding (red) sequence stretches and moderately binding HBMs (yellow) are highlighted in the respective structure of the proteins (C-I-TASSER model of S protein monomer [1]; PDBs: 6W37 (protein 7a), 6M18 (ACE2); Swiss-model: O15393 (TMPRSS2). Where the heme-binding affinity could not be determined, HBMs are also marked in yellow.

Supplementary Materials

Supplementary Tables

Table S1. Original evidence for the depicted connections for "Immune Response – Inflammation".

Source	Relation	Target	Original evidence	PMID	Resource
COVID-19	Increases	IL-1β	When **COVID-19** infects the upper and lower respiratory tract it can cause mild or highly acute respiratory syndrome with consequent release of pro-inflammatory cytokines, including **interleukin (IL)-1β** and IL-6.	32171193	COVID-19 KG
Heme	Increases	IL-1β	**Heme** activates macrophages inducing the production of TNF, KC (Figueiredo et al., 2007), **IL-1β** (unpublished), and LTB4.	24904418	Heme KG
COVID-19	Increases	IL-6	When **COVID-19** infects the upper and lower respiratory tract it can cause mild or highly acute respiratory syndrome with consequent release of pro-inflammatory cytokines, including interleukin (IL)-1β and **IL-6.**	32171193	COVID-19 KG
Heme	Increases	IL-6	Likewise, **heme** and FeNTA treatment causes the induction of the M1 markers MHC II, CD86, CD14, TNFα, **IL-6**, and IL1β and a decrease in the M2 markers CD206, IL-10, and Arginase-1 (the last with FeNTA only) in M0 BMDMs (Figure 3A; supplemental Figures 5, 6A, and 7).	26675351	Heme KG
COVID-19	Increases	TNFα	Initial plasma IL1B, IL1RA, IL7, IL8, IL9,	31986264	COVID-19 KG

			IL10, basic FGF, GCSF, GMCSF, IFNγ, IP10, MCP1, MIP1A, MIP1B, PDGF, **TNFα**, and VEGF concentrations were higher in both ICU patients and non-ICU patients than in healthy adults (appendix pp 6–7).		
Heme	Increases	TNFα	On the other hand, **TNF** secretion induced by **heme** is essential for the activation of the programmed necrotic cell death pathway, which is denominated necroptosis.<br>Likewise, **heme** and FeNTA treatment causes the induction of the M1 markers MHC-II, CD86, CD14, **TNFα**, IL-6, and IL1β and a decrease in the M2 markers CD206, IL-10, and Arginase-1 (the last with FeNTA only) in M0 BMDMs (Figure 3A; supplemental Figures 5, 6A, and 7).	24904418<br>26675351	Heme KG<br>Heme KG
TNFα	Increases	TLR4	Heme also induces tumour necrosis factor (**TNF**) secretion in monocyte/macrophages through **TLR4** and the adaptor molecule, MYD88/.	25307023	Heme KG
Heme	Increases	TLR4	**Heme** also induces tumour necrosis factor (TNF) secretion in monocyte/macrophages through **TLR4** and the adaptor molecule, MYD88/.	25307023	Heme KG
TLR4	Increases	MyD88	The **TLR4** activates two distinct pathways: **MyD88**	24904418	Heme KG

			and TRIF. In macrophages, heme induces a biased MyD88 activation and the secretion of the pro-inflammatory cytokines TNF and KC.		
MyD88	Increases	NF-κB	Only under protein-free conditions did we observe a limited heme-induced TNF-alpha response in cultured macrophages, which was triggered via signaling of the classical TLR4–**MyD88**–TRIF pathway of **NF-kB** activation.	29610666	Heme KG
NF-κB	Increases	IL-6	The MyD88-dependent pathway leads to the activation of mitogen-activated protein kinases (MAPKs) and **NF-κB** (nuclear factor kappa-light-chain-enhancer of activated B cells) transcription factors inducing the expression of inflammatory cytokines such as TNF, **IL-6**, IL-1β, and KC	24904418	Heme KG
COVID-19	Increases	IL-8	Initial plasma IL1B, IL1RA, IL7, **IL8**, IL9, IL10, basic FGF, GCSF, GMCSF, IFNγ, IP10, MCP1, MIP1A, MIP1B, PDGF, TNFα, and VEGF concentrations were higher in both ICU patients and non-ICU patients than in healthy adults (appendix pp 6–7).	31986264	COVID-19 KG
Heme	Increases	IL-8	Incubation of neutrophils with 4 mM **hemin** resulted in a 2-fold increase in **CXCL8** mRNA expression	28716864	Heme KG

			and in a significant increase in released and cell-associated levels of IL-8 protein compared with the untreated controls or A1AT-treated cells (Fig. 7A–C).		
COVID-19	Increases	CD14	Although patients had higher **sCD14** levels than healthy people, there were no significant differences between the severe and mild groups (Fig. 1b).	32203186	COVID-19 KG
Heme	Increases	CD14	Likewise, **heme** and FeNTA treatment causes the induction of the M1 markers MHC-II, CD86, **CD14**, TNFα, IL-6, and IL1β and a decrease in the M2 markers CD206, IL-10, and Arginase-1 (the last with FeNTA only) in M0 BMDMs (Figure 3A; supplemental Figures 5, 6A, and 7).	26675351	Heme KG
COVID-19	Increases	IL-10	Significantly high blood levels of cytokines and chemokines were noted in patients with **COVID-19** infection that included IL1-β, IL1RA, IL7, IL8, IL9, **IL10**, basic FGF2, GCSF, GMCSF, IFNγ, IP10, MCP1, MIP1α, MIP1β, PDGFB, TNFα, and VEGFA. Some of the severe cases that were admitted to the intensive care unit showed high levels of pro-inflammatory cytokines including IL2, IL7, **IL10**, GCSF, IP10, MCP1, MIP1α, and TNFα that are reasoned to promote disease severity.	32113704	COVID-19 KG

			Initial plasma IL1B, IL1RA, IL7, IL8, IL9, **IL10**, basic FGF, GCSF, GMCSF, IFNγ, IP10, MCP1, MIP1A, MIP1B, PDGF, TNFα, and VEGF concentrations were higher in both ICU patients and non-ICU patients than in healthy adults (appendix pp 6–7).	31986264	COVID-19 KG
Heme	Increases	IL-10	Interestingly, **heme** (10–30 μM) was also able to induce the secretion of **IL-10**, an anti-inflammatory cytokine.	23690673	Heme KG

Table S2. Original evidence for the depicted connections for "Immune Response – Complement system".

Source	Relation	Target	Original Evidence	PMID/doi	Resource
COVID-19	Increases	C3	**C3** activation products (C3 fragments C3a, C3b, iC3b, C3dg, and C3c) were detected by Western blotting in lung tissue of SARS-CoV MA15-infected mice, but not in control mice, as early as 1 day post infection (dpi) (Fig. 1B), confirming that SARS-CoV MA15 infection activates the complement pathway.	30301856	COVID-19 KG
Heme	Increases	C3	Furthermore, the incubation of serum or whole blood with **heme** induces deposition of activation fragments (C3b, iC3b, C3dg) of **complement component 3** (C3) at the surface of erythrocytes	26875449	Heme KG

COVID-19	Increases	Comple-ment activation	C3 activation products (C3 fragments C3a, C3b, iC3b, C3dg, and C3c) were detected by Western blotting in lung tissue of SARS-CoV MA15-infected mice, but not in control mice, as early as 1 day post infection (dpi) (Fig. 1B), confirming that SARS-CoV MA15 infection **activates the complement pathway**.	30301856	COVID-19 KG
Heme	Increases	Comple-ment activation	Hemolytic diseases are often accompanied by dysregulation and **overactivation of the complement system** [72–74], which may be induced by free extracellular **heme**.	26875449	Heme KG
COVID-19	Increases	Neutrophil activation	The common clinical manifestations of fever and cough in conjunction with laboratory results of progressively increasing neutrophil counts and leukocytopenia in serum samples taken from **COVID-19** patients at various stages of the illness indicates uncontrolled **neutrophil** extravasation and **activation**.	10.1101/2020.03.31.019216	COVID-19 KG
Heme	Increase	Neutrophil activation	**Heme-induced neutrophils activation** leads to extracellular traps (NETs) release through a mechanism dependent on ROS generation.	24904418	Heme KG
Heme	Increases	C5a	C3a and **C5a** anaphylatoxins, as well as the soluble membrane attack complex (sC5b9), are generated by incubation	26875449	Heme KG

			of **heme** with human serum or blood in vitro, via the alternative complement pathway).		

Table S3. Original evidence for the depicted connections for "Blood and coagulation system".

Source	Relation	Target	Original Evidence	PMID	Resource
COVID-19	Decreases	Albumin	**Albumin** concentrations were significantly lower in deceased patients than in recovered patients.	32217556	COVID-19 KG
Albumin	Decreases	Heme	Serum **albumin** (SA) can act as the heme scavenger by forming **heme**-SA complex [2, 4–8].	30324533	Heme KG
COVID-19	Increases	Ferritin	Levels of lactate dehydrogenase (LDH), concentrations of serum high-sensitivity C-reactive protein (hsCRP), **ferritin** and D-dimer levels were markedly higher in severe cases than moderate cases.	32217835	COVID-19 KG
Heme	Increases	Ferritin	In agreement with the electrical cell–substrate impedance sensing data described above, the proteome changes triggered by 10 μM **heme** were indicative of an adaptive response with prominent induction of HMOX1 and **ferritin** light (FTL) and heavy (FTH1) chains.	26794659	Heme KG
COVID-19	Decreases	Plasminogen activation	Figure 2. GO-term and KEGG pathway enrichment of up-regulated	32228226	COVID-19 KG

			expressed genes in BALF and PBMC of COVID-19 patients. [figure content]		
COVID-19	Decreases	Platelet count	Remarkable abnormalities in the CBC were detected on January 29, including increased neutrophils (10.67×10^9/L), leukocytes (11.73×10^9/L), and decreased lymphocytes (0.51×10^9 /L), and on February 4 decreased erythrocytes (2.40×10^{12}/L) and **platelets** (73×10^9 /L).	32196678	COVID-19 KG
Heme	Increases	Platelet aggregation	1 **Heme** induces **platelet** activation and **aggregation** through different pathways	26875449	Heme KG
Heme	Decreases	Fibrin	From another perspective, in vitro assays have demonstrated that **heme** can also bind to fibrinogen and decrease its thrombin-mediated cleavage, thus affecting the final common coagulation pathway and reducing **fibrin formation**, important in clotting (Figure 1).	26875449	Heme KG
Fibrin	Increases	Hemoglobin	During DIC, **fibrin** strands within the fibrin mesh formed could cut red blood cells, resulting in the formation of schistocytes (strongly deformed red blood cells or fragments of red blood cells) and the release of **hemoglobin**.	29956069	Heme KG
Hemoglobin	Increases	Heme	Toxicity of free **hemoglobin** is also caused by the release of cell-free **heme**, which produces lipid	27515135	Heme KG

			peroxidation and mitochondrial damage and increases the production of reactive oxygen species.		
Hemoglobin	Increases	Platelet aggregation	When RBCs are damaged by high shear in continuous flow ventricular assist devices, free **hemoglobin** induces **platelet aggregation**, contributing to high risk of thrombotic complications.	28458720	Heme KG

Table S4. Original evidence for the depicted connections for "Organ-specific diagnostic markers".

**Source**	**Relation**	**Target**	**Original Evidence**	**PMID**	**Resource**
Hemolysis	Increases	Heme	Free **heme** is generated by intra- and extravascular **hemolysis** or extensive cell damage.	24464629	Heme KG
Hemolysis	Increases	Bilirubin	LDH was strongly associated with markers normally elevated in either **hemolysis** or liver disease, including aspartate aminotransferase (AST) and direct and indirect **bilirubin**.	16291595	Heme KG
COVID-19	Increases	Bilirubin	Concentrations of alanine aminotransferase, aspartate aminotransferase, total **bilirubin**, alkaline phosphatase, and γ-glutamyl transpeptidase were markedly higher in deceased patients than in recovered patients.	32217556	COVID-19 KG
Heme	Increases	Bilirubin	The present findings demonstrate that the enzymatic mechanism	4334719	Heme KG

			catalyzing the conversion of **heme** to **bilirubin** in the liver is under hormonal control.		
Hemolysis	Increases	LDH	**LDH** was strongly associated with markers normally elevated in either **hemolysis** or liver disease, including aspartate aminotransferase (AST) and direct and indirect bilirubin.	16291595	Heme KG
COVID-19	Increases	LDH	Levels of lactate dehydrogenase (**LDH**), concentrations of serum high-sensitivity C-reactive protein (hsCRP), ferritin and D-dimer levels were markedly higher in severe cases than moderate cases.	32217835	COVID-19 KG

Table S5. Concordance between the common interactions and experimental data from Blanco-Melo et al. 2020 [2].

**Pathway**	**Gene symbol**	**Cell MMC2**	**Consistency MMC2**	**Cell MMC4**	**Consistency MMC4**
Immune system - Inflammation	NF-κB	0.4984	yes	0.6478	yes
	MyD88	0.199	yes	1.519	yes
	IL-6	2.390	yes	-0.517	no
	TLR4	0.1533	yes	1.917	yes
	IL-1β	1.036	yes	2.354	yes
	CD14	-0.4775	no	0.297	yes
	IL-10	0.539	yes	4.038	yes
	TNFα	1.333	yes	4.005	yes

Immune system - Complement system	C3	1.455	yes	0.760	yes
	C5	0.050	--	-1.392	--
Blood coagulation system	Hemoglobin	--	--	--	--
	Ferritin	0.047	yes	1.690	yes
	Albumin	0.0363	no	-0.1999	yes
Organ-specific diagnostic markers	LDHA	0.225	yes	0.658	yes
	LDHB	0.0	--	-0.738	no
	LDHC	0.043	yes	-0.979	no

Table S6. Characterization of potential heme-binding motifs within SARS-CoV-2 proteins (i.e., S protein and protein 7a) and human host cell proteins (i.e., ACE2 and TMPRSS2). Lines are highlighted according to the heme-binding affinity of the respective peptide (high (green) and moderate (yellow) heme-binding affinity, grey: heme-binding affinity could not be determined with UV/vis spectroscopy, red: not heme-binding)

General information			*Analytical characterization of peptides*			*Heme-binding properties*	
No.	Sequence	Position[a]	Mw[a] (Mw theor.) [g/mol]	HPLC t_R [min]	TLC R_f	K_D [µM]	Soret shift [nm]
			**S protein**				
**1**	FLGV**YYH**KN	F^{140} - N^{148}, extravirion	1039.637 (1138.581)	18.167[b]	0.479[f], 0.770[g]	0.96 ± 0.47[o]	~418
**2**	I**Y**SK**H**TPIN	I^{203} - N^{211}, extravirion	1071.649 (1070.576)	19.989[c]	0.288[f], 0.283[h],	7.87 ± 0.64[p]	~420
**3**	L**H**RS**Y**LTPG	L^{244} - G^{252}, extravirion	1042.599 (1041.561)	14.167[b]	0.342[f], 0.383[i],	26.09 ± 4.75[p]	~423

**Protein 7a**							
**4**	DGVK**H**V**Y**QL	D^{69} - L^{77}, extravirion	1057.612 (1056.561)	14.172[b]	0.250[f], 0.800[g]	n. b.	-
**5**	VK**H**V**Y**QLRA	V^{71} - A^{79}, extravirion	1142.685 (1141.613)	13.133[b]	0.130[j], 0.120[f]	n. sat.	~419
**ACE2**							
**6**	PL**Y**E**H**L**H**A**Y**	P^{235} - Y^{243}, extracellular domain	1141.605 (1140.561)	17.750[b]	0.149[h], 0.831[k]	4.04 ± 1.20[p]	~415
**7**	LTA**HH**EMG**H**	L^{370} - H^{378}, extracellular domain	1131.542 (1130.466)	17.360[c]	0.321[h], 0.831[k]	21.45 ± 1.54[p]	~420
**8**	SFIR**YY**TRT	S^{511} - T^{519}, extracellular domain	1205.676 (1204.624)	17.900[b]	0.120[j], 0.750[l]	0.60 ± 0.33[p]	~424
**9**	QAAK**H**EGPL	Q^{531} - L^{539}, extracellular domain	949.525 (948.502)	19.834[c]	0.666[g], 0.233[l]	n. b.	-
**10**	AMRQ**Y**FLKV	A^{650} - V^{658}, extracellular domain	1154.654 (1153.632)	15.131[d]	0.881[k], 0.783[l]	5.01 ± 0.78[p]	~432
**TMPRSS2**							
**11**	C(Me)VRL**Y**GPNF	C^{148} - F^{156}, extracellular domain	1081.615 (1080.527)	14.683[d]	0.242[g], 0.842[l]	n. b.	-
**12**	ILQV**Y**SSQR	I^{157} - R^{165}, extracellular domain	1092.592 (1091.598)	15.759[b]	0.385[g], 0.760[l]	n. sat.	~426 nm
**13**	RKSW**H**PVC(Me)Q	R^{165} - Q^{173}, extracellular domain	1153.629 (1152.271)	13.408[b]	0.182[g], 0.189[m]	33.44 ± 0.89[p]	~421 nm
**14**	RDMG**Y**KNNF	R^{186} - F^{194}, extracellular domain	1043.551 (1142.518)	12.287[b]	0.192[g], 0.436[l]	0.94 ± 0.38[p]	~422 nm

**15**	KNNF**Y**SSQG	K^{191} - G^{199}, extracellular domain	1043.488 (1042.472)	19.297[c]	0.130[j], 0.435[n]	n. sat.	~422 nm
**16**	KKL**YH**SDAC(Me)	K^{223} - C^{231}, extracellular domain	1077.615 (1076.517)	19.235[c]	0.365[g], 0.151[l]	n. sat.	~420 nm
**17**	KVIS**H**PN**Y**D	K^{330} - D^{338}, extracellular domain	1071.553 (1070.540)	19.777[c]	0.721[g], 0.106[h]	7.31 ± 0.59[p]	~419 nm
**18**	S**H**PN**Y**DSKT	S^{333} - T^{341}, extracellular domain	1047.522 (1046.467)	14.999[e]	0.451[g], 0.113[l]	n. b.	-
**19**	C(Me)AKA**Y**RPGV	C^{465} - V^{473}, extracellular domain	977.575 (976.501)	19.748[c]	0.260[k], 0.566[l]	n. sat.	~418 nm
**20**	TDWI**Y**RQMR	C^{481} - V^{489}, extracellular domain	1267.620 (1266.618)	14.254[d]	0.380[k], 0.717[l]	5.77 ± 0.89[p]	~420 nm

[a]Mass peaks were detected as $[M+H]^+$. [b]HPLC conditions were as follows: water with 0.1% TFA (eluent A), acetonitrile with 0.1% TFA (eluent B): 10% - 40% eluent B in 30 min. [c]HPLC conditions were as follows: water with 0.1% TFA (eluent A), acetonitrile with 0.1% TFA (eluent B): 0% - 40% eluent B in 40 min. [d]HPLC conditions were as follows: water with 0.1% TFA (eluent A), acetonitrile with 0.1% TFA (eluent B): 15% - 45% eluent B in 30 min. [e]HPLC conditions were as follows: water with 0.1% TFA (eluent A), acetonitrile with 0.1% TFA (eluent B): 0% - 30% eluent B in 30 min. [f]The following TLC system was used: pyridine/ethyl acetate/acetic acid/water (5:5:1:3, v/v). [g]The following TLC system was used: water/methanol/25% ammonia (10:90:25, v/v). [h]The following TLC system was used: 25% ammonia/n-propanol (3:7, v/v). [i]The following TLC system was used: methanol/acetic acid (10:15, v/v). [j]The following TLC system was used: acetonitrile/water/TFA (50:50:0.1, v/v). [k]The following TLC system was used: water/methanol/25% ammonia (25:75:15, v/v). [l]The following TLC system was used: pyridine/ethyl acetate/acetic acid/water (36:36:7:21, v/v). [m]The following TLC system was used: water/methanol/25% ammonia (75:25:15, v/v). [n]The following TLC system was used: hexan/ethyl acetate (8:2, v/v). [o]K_D value was determined according to the best fit, suggesting a stoichiometry of 1:2 peptide to heme. [p]K_D values were determined according to the best fit, suggesting a stoichiometry of 1:1 peptide to heme. HPLC: high-performance liquid chromatography, K_D: dissociation constant, Mw: molecular weight, n. b.: no binding, n. sat.: not saturated, R_f: retention factor, TLC: thin-layer chromatography, t_R: retention time.

Supplementary References

1. Zhang, C.; Zheng, W.; Huang, X.; Bell, E.W.; Zhou, X.; Zhang, Y. Protein structure and sequence reanalysis of 2019-nCoV genome refutes snakes as its intermediate host and the unique similarity between Its spike protein Insertions and HIV-1. *J. Proteome Res.* **2020**, *19*, 1351–1360.

2. Blanco-Melo, D.; Nilsson-Payant, B.E.; Liu, W.-C.; Uhl, S.; Hoagland, D.; Møller, R.; Jordan, T.X.; Oishi, K.; Panis, M.; Sachs, D.; et al. Imbalanced host response to SARS-CoV-2 drives development of COVID-19. *Cell* **2020**, *181*, 1036-1045.e9.
